# Polycyclic Aromatic Hydrocarbons in Electrocautery Smoke during Peritonectomy Procedures

**DOI:** 10.1155/2012/929053

**Published:** 2012-05-22

**Authors:** Sara Näslund Andréasson, Haile Mahteme, Bo Sahlberg, Helena Anundi

**Affiliations:** ^1^Department of Surgical Sciences, Uppsala University Hospital, 751 85 Uppsala, Sweden; ^2^Department of Occupational and Environmental Medicine, Uppsala University Hospital, Ulleråkersvägen 38-40, 751 85 Uppsala, Sweden

## Abstract

*Objective*. This study identified and quantified polycyclic aromatic hydrocarbons (PAHs) in electrocautery smoke during 40 peritonectomy procedures and investigated any correlations and/or differences between levels of PAHs and perioperative variables. *Methods*. PAHs were measured in personal and stationary sampling by 40 mm Millipore cassettes, for adsorption of both gaseous and particle-bound PAHs. *Results*. All 16 USEPA priority pollutant PAHs were detected during peritonectomy procedures, naphthalene being the most abundant. For the only two PAHs with Swedish occupational exposure limits (OELs), benzo[a]pyrene and naphthalene, limits were never exceeded. Amount of bleeding was the only perioperative variable that correlated with levels of PAHs. *Conclusions*. Low levels of PAHs were detected in electrocautery smoke during peritonectomy procedures, and an increased amount of bleeding correlated with higher levels of PAHs. For evaluation of long-term health effects, more studies are needed.

## 1. Introduction

The monopolar electrocautery (i.e., electrosurgical) device is an essential surgical tool that is used to cut through tissue and coagulate blood vessels [1] and thereby reduce blood loss and operative time. However, the smoke produced by the use of the electrocautery device is often considered to have an unpleasant smell and irritates the airways of the surgeons and the operating room staff [2–4]. Electrocautery smoke has been shown to contain considerable amounts of ultrafine particles (UFPs) [5, 6], indicating that the smoke may be potentially harmful [7]. The relationship between UFPs and polycyclic aromatic hydrocarbons (PAHs) has not yet been established. Still, some suggest that PAHs are often adsorbed to particles [8, 9], especially those PAHs of higher molecular mass or with five fused aromatic rings or more. PAHs with lower mass are present in the vapor phase [10]. There are over 200 PAHs, and they are primarily the result of incomplete combustion of organic material [10]. The International Agency for Research on Cancer (IARC) classifies PAHs into different groups depending on carcinogenicity [11]. Several PAHs are carcinogenic in animal studies and may also be carcinogenic to humans. Today, the most common site of PAH-caused cancer is the lung [10].

Electrocauterization is an essential part of almost all surgical treatments, especially when treating peritoneal carcinomatosis (PC). PC is a fatal condition without extensive surgical treatment, that is, peritonectomy combined with hyperthermic intraperitoneal chemotherapy (HIPEC) [12]. However, the use of the electrocautery device during peritonectomies produces a large amount of smoke and UFPs [6]. As PAHs are a product of combustion [10] and may adsorb to UFPs [8, 9], it is likely that electrocautery smoke also contains PAHs. Studies of the amount of PAHs in the electrocautery smoke from surgical procedures are limited. The primary aim of this study was to identify and quantify the United States Environmental Protection Agency's (USEPA) 16 priority pollutant PAHs in electrocautery smoke during peritonectomy procedures, and the secondary aim was to study any correlations and/or differences between levels of PAHs and perioperative variables (diagnosis, length of surgery, amount of bleeding, peritoneal cancer index (PCI), type of anaesthesia, and type of chemotherapy).

## 2. Methods

### 2.1. Description of Study Participants and Study Site

From 2009 to 2011, personal and stationary samplings of PAHs in electrocautery smoke were performed during a total of 40 peritonectomy procedures at Uppsala University Hospital. The regional ethics committees approved the study.

The 40 peritonectomies from which PAHs were collected included 20 female and 20 male patients suffering from PC from pseudomyxoma peritonei (PMP) (*n* = 22), colorectal cancer (CRC) (*n* = 11), appendiceal cancer (*n* = 5), and ovarian cancer (*n* = 2). All patients were anesthetized, either with a combination of tiopental (Pentothal), rokuroniumbromid (Esmeron), fentanyl, and isoflurane (PEF/ISO) (*n* = 32), or a combination of propofol (Diprivan), rokuroniumbromid (Esmeron), fentanyl, and isoflurane (DEF/ISO) (*n* = 8). All but three patients received HIPEC: cisplatin/doxorubicin (*n* = 17), oxaliplatin/irinotecan (*n* = 13), or mitomycin C (*n* = 7).

**Figure 1:**
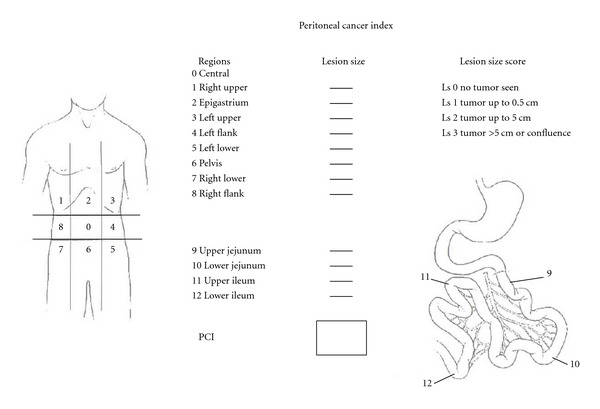
The peritoneal cancer index (PCI). This index combines size and distribution parameters to obtain a numerical score. The lesion size scores (0–3) in each of the abdominopelvic regions (*n* = 13) are summed to give the peritoneal cancer index (range 0–39) [13]. This graphic is published by permission from Dr. P. H. Sugarbaker.

Peritonectomy was performed as described by Sugarbaker [12]. The peritonectomy procedure is a surgical intervention with the aim of removing disseminating cancers, that is, PC, from the abdomen [12]. Tumor load was recorded according to the peritoneal cancer index (PCI) (range 1–39) (Figure 1) [13], in order to assess tumor load in the abdominal cavity. PCI is calculated by summing lesion size scores (0–3) within the abdominopelvic regions (0–12) (maximum 3×13 = 39).

The electrocautery generator used during this study was a VIO 300 D (ERBE, SN 11260962, Elektromedizin, Tübingen, Germany), set on a dry cut at a high voltage of 200/300 W.

When all macroscopic tumors have been removed, HIPEC is distributed within the open abdomen for 30–90 minutes to annihilate any remaining microscopic tumors [12], at an abdominal temperature of 41.5–43°C [14]. The chemotherapeutic drugs are circulated with the help of a roller pump, and a heat exchanger is connected to the circuit [13, 15] to warm the drugs. Additionally, two smoke evacuators are placed towards the opening of the plastic sheet during the entire treatment, to remove any vapors from the heated chemotherapeutic drugs.

Descriptive statistics regarding length of surgery, PCI, and amount of bleeding for the 40 peritonectomy procedures are presented in Table 1. In one procedure, data for PCI and amount of bleeding are missing.

### 2.2. Environmental Sampling

Both personal and stationary samplings of PAHs in electrocautery smoke were performed. Samplings started at the beginning of the surgery and ended when the abdomen was surgically closed. All 40 measurements were performed in the same operating room, and the same OR staff assisted at all sampling occasions.

The operating room was 46 m^2^ and had 20 air changes per hour. Air quality parameters, such as relative air humidity, temperature, and carbon dioxide (CO_2_), were continuously measured in the operating theatre during the procedures, using a Q-Trak instrument (Q-Trak, IAQ Monitor, model 8550, TSI Incorporated). During all peritonectomies, the mean relative air humidity in the operating room was 23.0%, the temperature was 21.7°C, and the average CO_2_ was 485 ppm.

#### 2.2.1. Personal Sampling

Personal sampling was performed using a 40 mm Millipore cassette fixed near the surgeon's breathing zone. The cassette contained XAD adsorbent for adsorption of gaseous PAHs and a glass-microfiber Munktell filter grade MG 160 for particle-bound PAHs. The cassette also contained an internal standard (PAH-Mix 9 deuterated “XA20950902CY” mix) from LGC Standards AB (Borås, Sweden). The cassette was connected to an SKC AirChek 5000 XR pump (SKC Inc., PA, USA) with an airflow of 4.2 litres/min [16].

#### 2.2.2. Stationary Sampling

Stationary sampling of PAHs was performed using a 20 mm wide smoke evacuating hose, connected to a Smoke Plume Evacuation System IES 2 (ERBE, Type nr 10321-000, App nr C-2046, Elektromedizin, Tübingen, Germany) with a set efficiency of 100%. A minor cut was made in the hose in order to insert and attach the tube to the filter cassette, Millipore (40 mm), which collected smoke particles and gases evacuated five cm from the tip of the electrocautery device. The cut in the hose was sealed with tape to prevent leakage of the collected smoke.

### 2.3. Sample Analyses

The samples were sent to Alcontrol Laboratories (Linköping, Sweden) for analysis. Prior to the analysis, XAD mass and the filter from the sample container were transferred to a test tube and extracted in an ultrasonic bath for 10 minutes. The extraction was repeated three times with a total of 20 mL of dichloromethane (DKM), which was combined in a round flask. The extract was then roto evaporated and transferred to a test tube, was evaporated under nitrogen gas and heat (30°C), and then was ready for analysis [16].

Samples were analysed by gas chromatography (HP 6890) using a DB5-MS column (3 m × 0.25 mm, 0.25 micron stationary phase with (5% phenyl)-methylpolysiloxane) from Agilent J & W. Helium was used as the carrier gas with a constant flow of 1.5 mL/min. The injection temperature was set to 280°C, and the injection volume was 1 microliter. The oven program was set to 60°C for 1 min and ramped at 8°C/min to 310°C. The ionization method on the mass spectrometer (HP 5973) included electron impact of interface temperature 310°C and ion source temperature at 230°C. The Quadrupole temperature was set to 150°C, and the selected ion-monitoring (SIM) mode was used. Identification and quantification were carried out against calibration standards and with known concentrations using the internal standard method [16].

### 2.4. Statistical Analyses

Any correlations between PAHs from all 40 peritonectomy procedures and the perioperative variables (length of surgery and amount of bleeding) were determined using Spearman's rank correlation coefficient. Furthermore, a multiple regression was executed to establish possible predictors for the amount of bleeding among the PAHs. Spearman's rank correlation coefficients were calculated for 20 of the 40 peritonectomies, measured on separate filters, to detect any correlations between PAHs with single procedures' diagnosis, PCI, length of surgery, amount of bleeding, type of anaesthesia, and type of chemotherapy. Additionally, Mann-Whitney *U* test was used to look for differences between PAHs in PMP versus CRC, PAHs in cisplatin/doxorubicin versus oxaliplatin/irinotecan, and PAHs in PCI < 19 versus ≥20 (CRC). A 2-sided *P* value of less than 0.05 was considered statistically significant. Statistical analyses were performed with Statistica 10.0 (StatSoft, Inc., Tulsa, OK, USA).

## 3. Results

### 3.1. Identification and Quantification of PAHs

All 16 PAHs in electrocautery smoke were detected, but not in all samples. In the 40 peritonectomy procedures, the most abundant compound was naphthalene, detected in all but one sample. The most abundant PAHs, apart from naphthalene, were phenanthrene (93%), fluorene (63.3%), acenaphthene (40%) and acenaphthylene (36.7%) in personal samples. In stationary sampling, acenaphthylene was detected in 93.3%, phenanthrene in 90%, acenaphthene in 90%, and fluorene in 83.3% of the samples. Geometric means (GM) and geometric standard deviations (SDs) of PAHs (ng/m^3^) for all 40 peritonectomy procedures are presented in Table 2.

### 3.2. Correlations between PAHs and Perioperative Variables

There was no correlation between PAHs and length of surgery in the 40 peritonectomy procedures. However, both personal and stationary sampling of PAHs and amount of bleeding correlated to some extent (Table 3), but possible predictors for the amount of bleeding among the PAHs were not found. Acenaphthene and fluorene correlated with the amount of bleeding in personal sampling, and benzo[a]anthracene, benzo[a]pyrene, benzo[b]fluoranthene, chrysene/triphenylene, acenaphthylene, anthracene, benzo[ghi]perylene, phenanthrene, fluoranthene, fluorene, naphthalene, and pyrene correlated in stationary samplings.

Diagnosis, PCI, length of surgery, type of anesthesia, and type of chemotherapy did not correlate with PAHs within the grouping of 20 procedures, sampled separately.

### 3.3. Differences between PAHs and Perioperative Variables

A statistical difference could only be found between phenanthrene in PMP versus CRC (*P* = 0.04) and phenanthrene in cisplatin/doxorubicin versus oxaliplatin/irinotecan (*P* = 0.04), in personal sampling. PAHs in PCI < 19 versus ≥20 (CRC) showed no statistical differences.

## 4. Discussion

All 16 PAHs could be detected in both personal and stationary samplings, but the levels of the most carcinogenic substances were low. However, higher levels of carcinogen PAHs were detected in single procedures, indicating that higher cumulative amounts were being inhaled by surgeons and operating room staffs. Naphthalene was the most common PAH in both personal and stationary samplings of this study. None of the most abundant compounds (naphthalene, acenaphthylene, acenaphthene, phenanthrene, and fluorene) have been proven to be carcinogenic. However, naphthalene is stated to be a possible human carcinogen [10, 11], and single, high doses have caused bronchiolar necrosis in animals. Naphthalene is also embryotoxic to mice and rats and causes cataract in mouse eyes, and phenanthrene may induce skin reactions after dermal application [10]. Several sources of both known and possible PAH carcinogens surround humans every day [17–23]; some are probable causes of cancers [23–25], and some probably cause other diseases [26–29]. An increase in cancer has been noted when humans have been exposed to several PAH-containing mixtures. However, it is difficult to say whether the increase depends on the PAHs exclusively, or if the mixtures include other carcinogenic compounds [10]. Additionally, PAHs may affect fetal growth [30, 31].

Interestingly, significant correlations were demonstrated between PAHs and amount of bleeding, within both personal and stationary samplings. This has not been reported earlier. Blood consists of blood cells, blood plasma (90% water containing plasma proteins and electrolytes: sodium chloride, potassium, calcium, magnesium salts, and phosphates), and other components [32]. It is possible that some blood components are affected by the heat from the electrocautery device when coagulating a blood vessel and produce PAHs. In addition, the levels of PAHs in patients' blood before surgery could differ, for example, depending on whether they are smokers or not [22, 23].

A statistical difference could only be found in personal sampling between phenanthrene in PMP versus CRC, and between phenanthrene in cisplatin/doxorubicin versus oxaliplatin/irinotecan. Most probably, this difference depends on skewness within the groups compared due to limited observations (*n* = 14, PMP versus *n* = 4 CRC, and *n* = 15, cisplatin/doxorubicin versus *n* = 5 oxaliplatin/irinotecan). Unfortunately, this is one of the consequences of studying peritonectomy procedures consecutively, instead of sorting them into groups of diagnosis, PCI, length of surgery, and so forth, which may vary considerably. Among these procedures, there may also be a problem of forming sufficiently large groups for statistical analysis. Nevertheless, this is the first study of its kind, and the main purpose of the investigation was to identify and quantify the 16 USEPA-recommended PAHs in electrocautery smoke. Finding single high levels of PAHs is, of course, important because of their known or possible carcinogenicity [10, 11], but it may be even more interesting to report cumulative levels of PAHs for those who are exposed in their everyday work. In this study, mixtures of PAHs were present that are known to increase the risk of cancer [10].

Among the most abundant PAHs in this study, naphthalene has the lowest molecular mass, with two fused aromatic rings, and acenaphthylene, acenaphthene, phenanthrene, and fluorene follow with three rings. The heavier PAHs, with more aromatic rings, are not represented in this group. PAHs may adsorb to particles [8, 9], especially PAHs with five rings or more, whilst others vaporize [10]. Yamasaki et al. [33] found that PAH increased with ambient air temperature [33]. In an iron foundry (at a PAH source temperature of 600–700°C) of the PAHs in the vapor phase, 70% were four to seven rings [34]. When using the electrocautery device, tissue temperature may reach 150–400°C [35].

There are only occupational exposure limits (OELs) for two PAHs in Sweden: benzo[a]pyrene 2 *μ*g/m^3^ LLV (*level limit value*: an occupational exposure limit value for exposure during one working day) and 20 *μ*g/m^3^ STV (*short-term value*: reference period of 15 minutes); naphthalene 50 mg/m^3^ LLV and 80 mg/m^3^ STV [36]. In the USA, the permissible exposure limit (PEL) for benzo[a]pyrene is 0.2 mg/m^3^ and 50 mg/m^3^ for naphthalene. Mean values of the results of the samplings of benzo[a]pyrene and naphthalene in this study were well below the Swedish OELs. Moreover, no single value exceeded the limits of these PAHs.

The strength of this study is the homogeneity under which the samplings were executed. During all 40 samplings, the same method and the same operating room have been used, and the same personnel have been present. Additionally, stationary samplings have been very precise due to the possibility of attaching the filter within the smoke evacuation hose. The smoke that was sucked into the hose was collected 5 cm from the electrocautery device, which should concentrate and enhance the amount of smoke for analysis. Consequently, personal samplings collected fewer kinds and lesser amounts of PAHs than the stationary samplings, perhaps as the personal filter was farther from the source of the electrocautery smoke. This is the first study to identify and quantify USEPA's 16 priority pollutant PAHs in electrocautery smoke during peritonectomy procedures. Regardless of the duration of the peritonectomy procedures in this study, low levels of PAHs were sampled. Consequently, the hazard of adverse effects from inhaling PAHs should be minimal. Although long-term exposure to PAHs could lead to high cumulative levels in surgeons and operating room staffs, one should also consider the simultaneous exposures of particles, PAHs, and volatile organic compounds, and that there may be synergistic and additive effects. More studies are needed to evaluate the level, and the possible risk, of PAH exposure in the operating room. Larger and selected study groups seem to be necessary to increase the chance of significant findings.

## 5. Conclusions

Low levels of PAHs were detected in electrocautery smoke during peritonectomy procedures. Naphthalene, which is considered to be a possible carcinogen, was the most abundant PAH in both personal and stationary samplings. Only the amount of bleeding correlated with PAHs, which is interesting in a larger perspective as the electrocautery device is essential in almost all surgical interventions.

## Figures and Tables

**Table 1:** Perioperative variables: Length of surgery, PCI, and bleeding in 40/39 peritonectomy procedures.

Perioperative variables	Median	Lower quartile	Upper quartile
Length of surgery (h : min) (*n* = 40)	10 : 14	7 : 08	11 : 33
PCI (score) (*n* = 39)	20	11	30
Bleeding (mL) (*n* = 39)	500	100	900

h: hours, min: minutes, *n*: number, PCI: peritoneal cancer index, and mL: milliliter.

**Table 2:** GM and GSD (ng/m^3^) of PAH in 40 peritonectomy procedures.

PAH	GM	GSD
Benzo[a]anthracene P/S	0.14/0.14	±2.68/±2.51
Benzo[a]pyrene P/S	0.13/0.16	±2.43/±2.92
Benzo[b]fluoranthene P/S	0.16/0.21	±3.20/±3.83
Benzo[k]fluoranthene P/S	0.14/0.16	±3.14/±3.06
Chrysene/triphenylene P/S	0.15/0.34	±3.31/±6.00
Dibenzo[a,h]anthracene P/S	0.11/0.13	±1.88/±2.95
Indenol[1,2,3-cd]pyrene P/S	0.12/0.12	±2.25/±1.96
Acenaphthene P/S	0.49/6.24	±8.46/±5.64
Acenaphthylene P/S	0.34/14.63	±5.87/±5.71
Anthracene P/S	0.11/0.35	±1.94/±5.40
Benzo[ghi]perylene P/S	0.12/0.16	±2.15/±3.10
Phenanthrene P/S	4.07/6.27	±3.16/±5.17
Fluoranthene P/S	0.19/0.58	±3.99/±7.02
Fluorene P/S	0.90/5.18	±7.07/±6.15
Naphthalene P/S	63.41/178.66	±2.20/±9.32
Pyrene P/S	0.15/0.50	±3.18/±6.84

PAH: polycyclic aromatic hydrocarbon, P: personal sampling, S: stationary sampling, GM: geometric mean, GSD: geometric standard deviation.

**Table 3:** Spearman rank correlations between PAHs and bleeding.

PAH	Bleeding in 40 peritonectomies			
	Correlations	−95% CI	+95% CI	2-sided *P* value
Benzo[a]anthracene P/S	−0.147/**0.501**	−/0.155	−/0.728	ns/**0.05**
Benzo[a]pyrene P/S	−0.138/**0.596**	−/0.283	−/0.785	ns/**0.0006**
Benzo[b]fluoranthene P/S	−0.121/**0.475**	−/0.122	−/0.712	ns/**0.009**
Benzo[k]fluoranthene P/S	−0.111/0.116			
Chrysene/triphenylene P/S	−0.123/**0.549**	−/0.219	−/0.758	ns/**0.002**
Dibenzo[a,h]anthracene P/S	−0.167/0.324			
Indenol[1,2,3-cd]pyrene P/S	−0.116/0.086			
Acenaphthene P/S	**0.397**/0.348	0.028/−	0.662/−	**0.03**/ns
Acenaphthylene P/S	0.289/**0.499**	−/0.153	−/0.727	ns/**0.005**
Anthracene P/S	0.288/**0.634**	−/0.338	−/0.807	ns/**0.0002**
Benzo[ghi]perylene P/S	−0.122/**0.433**	−/0.071	−/0.686	ns/**0.01**
Phenanthrene P/S	0.061/**0.480**	−/0.129	−/0.715	ns/**0.008**
Fluoranthene P/S	0.081/**0.492**	−/0.145	−/0.723	ns/**0.006**
Fluorene P/S	**0.418**/**0.538**	0.053/0.204	0.676/0.751	**0.02**/**0.002**
Naphthalene P/S	0.320/**0.455**	−/0.098	−/0.700	ns/**0.01**
Pyrene P/S	0.163/**0.573**	−/0.251	−/0.772	ns/**0.001**

PAH: polycyclic aromatic hydrocarbons, P: personal sampling, S: stationary sampling, ns: not significant.
